# Nanodeformations of microcapsules: comparing the effects of cross-linking and nanoparticles†

**DOI:** 10.1039/d2ra04330k

**Published:** 2022-08-24

**Authors:** Ulrike Doering, Dmitry Grigoriev, Tino Riske, Andreas Fery, Alexander Böker

**Affiliations:** University of Potsdam, Institute of Chemistry Karl-Liebknecht-Str. 24-25 14476 Potsdam Germany; Fraunhofer Institute for Applied Polymer Research IAP Geiselbergstr. 69 14476 Potsdam Germany dmitry.grigoriev@iap.fraunhofer.de; Leibniz-Institute for Polymer Research Dresden e.V. Hohe Str. 6 01069 Dresden Germany

## Abstract

The mechanical properties of proteinaceous and composite microcapsules loaded with oil were measured by SFM and evaluated using the Reissner model. Comparison of the obtained results reveals significantly higher Young’s moduli of protein capsules due to intermolecular crosslinking. In contrast, conformational restrictions in composite microcapsules inhibit protein crosslinking leading to the reduction of their elasticity.

## Abstract 2

SFM results for protein and composite microcapsules are evaluated by the Reissner model. Protein capsules show higher Young’s moduli due to crosslinking, which is absent in composite capsules because of restrictions in the protein conformations.

## Graphical abstract

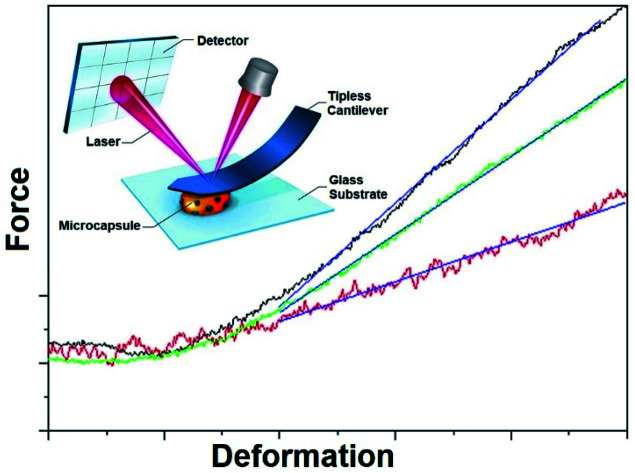

## Introduction

Encapsulation is an efficient way of protecting sensitive, especially biochemical, agents or controlling their release. Hence, microcapsules are widely used in different biomedical, food and cosmetics applications for the confinement of various bioactive agents such as nutrients,^1^ fragrances,^2^ pharmaceuticals^3^ and catalysts.^4^ The mechanical characteristics of the microcapsules are of key importance for many applications since they determine integrity, sensitivity and stability of capsules. In particular, such factors as the preparation method and the chemical composition of the capsules shell can significantly affect these characteristics.

Another important aspect of the capsules preparation and especially of their following application is a proper choice of the shell composition. In particular, in the composite capsules aimed at using in biomedical applications, most common inorganic materials frequently used in the nanoparticle (NP) form are calcium carbonate,^5^ calcium phosphate^6,7^ or silica.^8,9^ The physical characteristics of the microcapsules, including biocompatibility, permeability and mechanical strength can be controlled by the choice of colloids. On the other hand, biopolymers as capsules shell material possess a significant advantage in that they are adjustable stimuli-responsive, which enables a controlled release of loaded substances.^10–12^ Their biodegradability, natural abundance and low costs are further benefits.

As biological building blocks^13^ for capsule shells, proteins have also been thoroughly examined. With their structural and chemical versatility, amphiphilic character and emulsifying properties they offer various advantages.^14^ Suslick and Grinstaff developed a technique for the synthesis of protein microcapsules using ultrasound.^15^ The capsule formation is a result of two very fast subsequently occurring phenomena: protein adsorption and shell cross-linking.^15–18^ In our previous work^19^ we presented several experimental proofs pointing towards the formation of intermolecular S–S bonds in the capsule shell made of bovine serum albumin (BSA) and corresponding structural changes of the protein during the process. The preparation of new hybrid materials will be enabled by the combination of proteins and inorganic nanoparticles.^6,20–22^

In this paper, we compared the mechanical properties of oil loaded cross-linked protein microcapsules and non-cross-linked protein- and nanoparticle-stabilized composite microcapsules that were prepared in one-pot process using high-intensity ultrasound. Both capsule types with biocompatible and biodegradable shells potentially suitable for medical applications were investigated in aqueous conditions by nanocompression. The measurements were performed using a scanning force microscope (SFM) and the Young’s moduli were obtained from the recorded force–deformation curves within the small-deformation regime.

## Materials and methods

### Materials

BSA (>95%) was purchased from Alfa Aesar (Germany). Miglyol 812 was purchased from Sasol (Germany) and BisTris (≥99%) buffer solution was purchased from Carl Roth (Germany). The aqueous Ludox Cl suspension (aluminum oxide coated silica NPs with average size of 25 nm, 30% w/v) was purchased from Sigma Aldrich (Germany).

### Preparation of oil filled protein microcapsules

In a cylindrical vessel, 0.35 mL Miglyol was layered over 3.15 mL of a 5% w/v BSA solution. The used volume ratio of aqueous/organic phase was 9 : 1. A high-intensity ultrasonic horn with a tip diameter of 3 mm was placed at the oil–water interface. To maintain the temperature below 30 °C during ultrasonication, the vessel was positioned in an ice bath. The solutions were sonicated for 1 minute at an acoustic power of ∼200 W cm^−2^. Simultaneously, the solutions were mixed with a magnetic stirrer. The obtained microcapsules were dialyzed against distilled water with pH of 6.7 using a dialysis tube with a cutoff of 1000 kDa (Spectrum Labs Spectra/Por Dialysis Membrane Biotech CE) to remove residual chemicals and fragments of broken microcapsules. These microcapsules are denoted further as BSA-Miglyol microcapsules.

### Preparation of oil filled composite microcapsules

In the first step, the Ludox Cl NPs were pre-modified with BSA. For this purpose, a NP suspension (1% w/v) in distilled water (pH 6.7) was added dropwise to an equal volume of a BSA solution (5% w/v) in distilled water (pH 6.7). After multistep washing process in BisTris buffer solution (20 mM, pH = 7) and subsequent centrifugation at 14 000 rpm, the BSA modified NPs were redispersed with a BisTris solution. In a cylindrical vessel, Miglyol (1.4 mL) was layered over the BSA modified NP suspension (2.1 mL). A high-intensity ultrasonic horn with a tip diameter of 3 mm was placed at the oil–water interface. To maintain the temperature below 30 °C during ultrasonication, the vessel was positioned in an ice bath. The system was sonicated for 1 minute at an acoustic power of ∼200 W cm^−2^. Simultaneously, the system was mixed with a magnetic stirrer. The obtained microcapsules were dialyzed as well against distilled water with pH of 6.7 using a dialysis tube with a cutoff of 1000 kDa. These microcapsules are denoted further as BSA-Miglyol-Ludox Cl.

### Nanocompression measurements with SFM

The measurements were carried out in a BisTris buffer solution in a liquid measuring cell with a round glass object slide. To prevent a movement of the microcapsules during the measurement, an immobilization of the capsules on the glass surface is necessary. For this purpose, a few drops of a PEI solution (1 g L^−1^) were added and homogeneously distributed on the object slide that was cleaned and pre-treated with O_2_ plasma. After the evaporation of the solvent, a thin polymer film remains, on which a few droplets of the capsule dispersion were added. After short drying on air, the microcapsules are fixed on the surface of the object slide. The measuring cell was inserted in the SFM and filled with the BisTris buffer solution. The measurements were carried out in a climate-controlled laboratory at 22 °C. The nanocompression measurements were carried out with a SFM MFP-3D (Asylum Research, USA) combined with an inverted optical microscope (Zeiss, Germany). The used cantilever was HQ:NSC36/tipless/No Al (*k* = 2.0 N m^−1^, *f* = 180 kHz, manufacturer’s nominal values) from MikroMasch (Estonia). Two cantilevers of this type were used for the measurements. The spring constants were previously determined using the thermal method. The microcapsules BSA-Miglyol and BSA-Miglyol-Ludox Cl (2.6%) were measured with a cantilever with a spring constant of *k* = 3.91 N m^−1^ (*f* = 182.898 kHz). The microcapsules BSA-Miglyol-Ludox Cl (4.7%) were measured with a cantilever with a spring constant of 3.79 N m^−1^ (*f* = 182.412 kHz). The measuring rate was 1 μm s^−1^. The diameters of the examined capsules were determined before the measurements. Subsequently, the cantilever was brought into contact with the microcapsule and the measurement was started by deforming them and recording the force required for the corresponding deformation. Usually nanocompression measurements are performed by approaching the microcapsules with a glass microbead glued at a tipless cantilever known as colloidal probe.^23^ However, Glynos and colleagues have shown that such measurements for the determination of the Young’s modulus of microcapsules with thin polylactide shell were also possible without using this technique, just by means of a bare cantilever instead.^24^

**Fig. 1:**
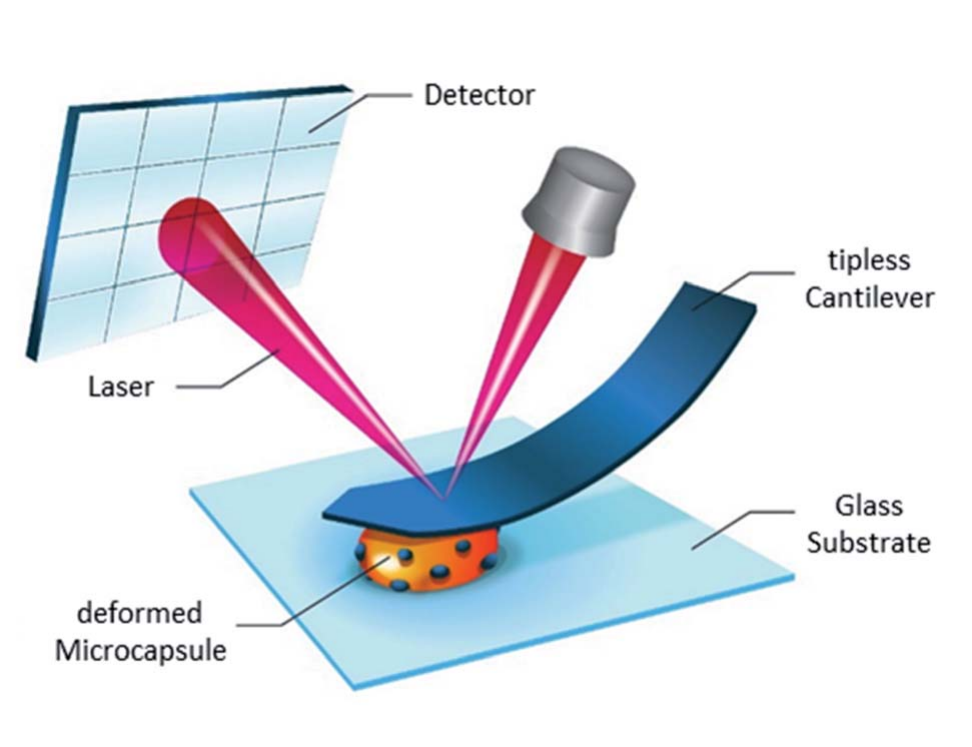
SFM indentation for Young’s modulus determination of composite microcapsules.

Since it turned out to be challenging to exert the load with the glass microbead at the poles of oil-loaded microcapsules without their slipping or moving them away, bare cantilevers were used to deform thin-shell microcapsules and calculate their Young’s modulus. Fig. 1 displays schematically this experimental procedure in the particular case of composite microcapsules.

## Results and discussion

**Fig. 2:**
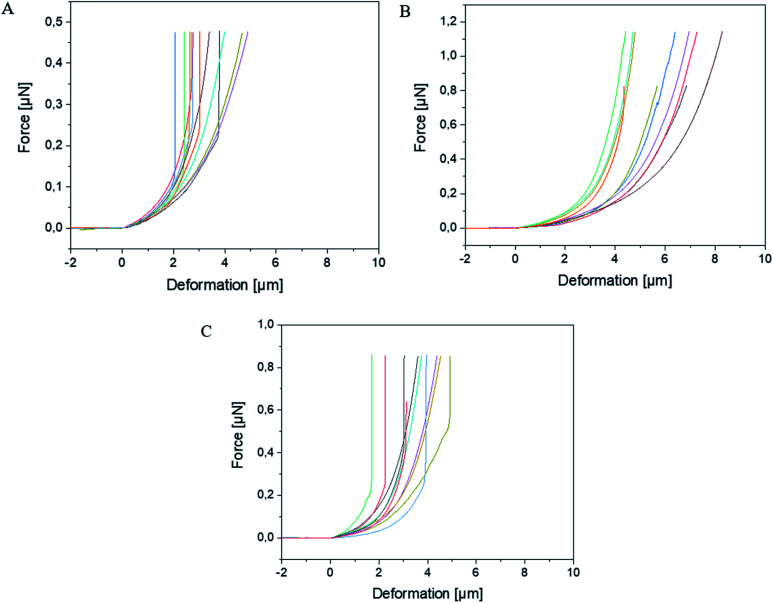
Force *versus* deformation curves of (A) BSA-Miglyol microcapsules, (B) composite BSA-Miglyol microcapsules with 2.6% Ludox Cl and (C) with 4.7% Ludox Cl in the shell. The different color lines in each diagram refer to the different measured capsules of the corresponding sample.

The experimental dependencies of force as function of deformation for different types of microcapsules are presented in Fig. 2.

**Fig. 3:**
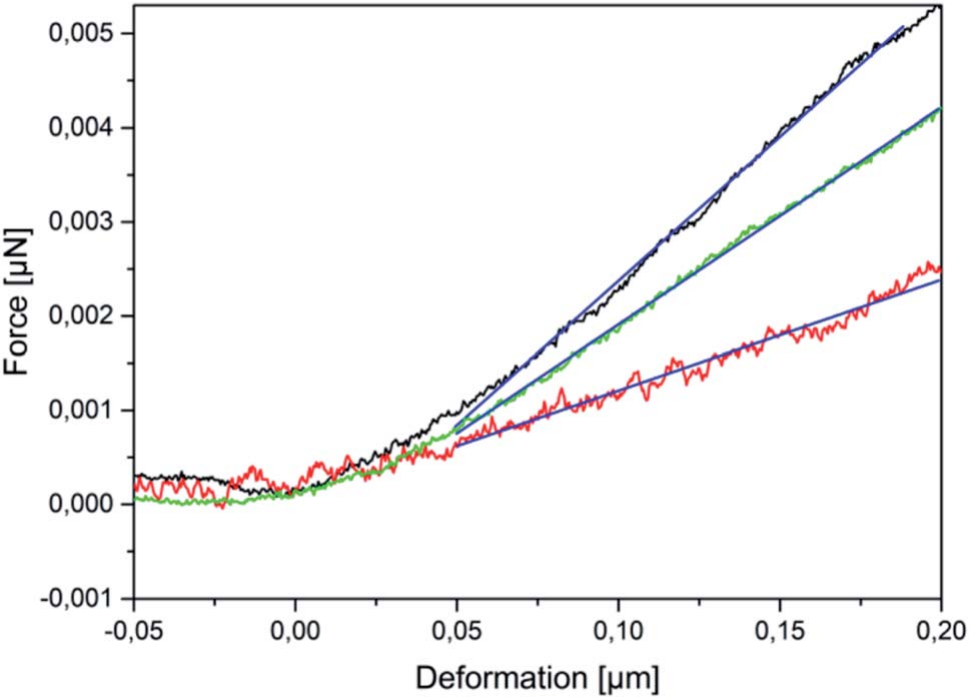
Force *versus* deformation curves in the small-deformation regime of BSA-Miglyol microcapsules (black), composite BSA-Miglyol microcapsules with 2.6% Ludox Cl (red) and with 4.7% Ludox Cl (green) in the shell.

A zero force baseline is observed during the approach before the contact with the sample. Upon further approaching, the cantilever contacts the sample. In the small deformation regime, the indentation increases linearly with applied force when the capsules are compressed (see Fig. 3). The microcapsules show elastic response in this regime. The initial linear part of the resulting curves is used for the determination of the mechanical properties of the microcapsules. Notable that the distance on the force–deformation curve between the first non-zero point and its abruptly increasing region is sufficiently smaller than the size of the investigated microcapsules. This phenomenon is caused by the non-horizontal tilted orientation of the cantilever and by the fact that the capsules under measurement were compressed not by its terminal part but by its first third towards the tip (see Fig. 1). Although the size of the investigated capsules was essentially larger than this distance with the length of 2 to 4 micrometers, their small deformation by the middle part of the cantilever was accompanied by the contact between its terminal part and the rigid glass surface on which the microcapsules are immobilized leading to the vertical increase of the measured force (see Fig. 2). Before every measurement with the SFM, microscopic images of the microcapsules were recorded for the determination of their sizes. The BSA-Miglyol microcapsules had an average size of 5.4 ± 1.1 μm and a shell thickness of 10 nm (determined by SFM, see Fig. S2†), while the composite oil filled microcapsules with 2.6% Ludox Cl in the shell possessed an average size of 7.6 ± 1.8 μm.^22^ An increase of the NP concentration to 4.7% resulted in a slight decrease of the average composite microcapsules size with 5.6 ± 1.8 μm.^22^ As the size of the largest component of the composite shell, silica nanoparticles, is, according to the manufacturer, about of 10 nm, the realistic estimation of the shell thickness can be of the order of 25 nm, taking into account the possible formation of small aggregates (like particle doublets and triplets) in the shell.

The BSA-Miglyol capsules exhibited a steeper initial slope in the small deformation regime of the force–deformation curve than the composite BSA-Miglyol microcapsules. In spite of hard NPs in composite shells, the conformational restrictions in BSA adsorbed on particles cause its inability for intermolecular crosslinking and hence lead to a lower stiffness of these shells than for cross-linked proteinaceous ones.^22^ However, the different slopes of the composite microcapsules show that their mechanical properties can be tailored by varying the concentration of the NPs as the slope increased with increasing Ludox Cl concentration.

To calculate the Young’s modulus of the microcapsules, the analytical solution of Reissner^25,26^ for small deformations of isotropically elastic thin-shell microcapsules can be applied, if the following conditions are satisfied: the ratio of shell thickness to radius should be smaller than 1/20 and a point-like load must be exerted at the poles of the capsules.^24–26^ The equation connecting the force and the deformation with the material and the geometric characteristics of the thin-shell microcapsules of a radius *R* and a shell thickness *h* is as follows:1
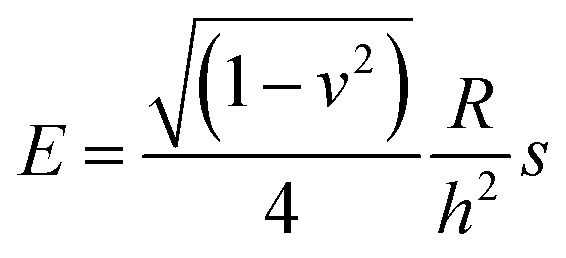
where *E* is the Young’s modulus and *ν* is the Poisson’s ratio, which was assumed as 0.5 due to BSA being an isotropic incompressible elastic material, while *s* is the slope of the initial linear part of the force deformation-curve. The Young’s moduli calculated with this equation are given in Table 1.

**Table 1:** Young’s moduli of BSA-Miglyol microcapsules and composite BSA-Miglyol microcapsules obtained by Reissner model. Size of each capsule type was taken as the mean of 12 measurements

Microcapsules	Young’s modulus [MPa]	Diameter [μm]
BSA-Miglyol	191 MPa ± 64	5.4 ± 1.1
BSA-Miglyol-Ludox Cl (2.6%)	29 MPa ± 7	7.6 ± 1.8
BSA-Miglyol-Ludox Cl (4.7%)	38 MPa ± 12	5.6 ±1.8

The BSA-Miglyol capsules exhibited the highest Young’s modulus with 191 MPa ± 64 MPa. A comparable system was described by Ye and coworkers, who used the colloidal probe SFM technique to determine the Young’s modulus of pea protein microcapsules.^27^ These capsules were also synthesized by sonication and due to the high content of cysteine, the formation was based on the cross-linking mechanism as well. Moreover, the diameters of the pea protein capsules are with 3–5 μm in a similar range as the BSA-Miglyol capsules. Nevertheless, the obtained average Young’s moduli were significantly lower with 0.58 to 2.35 MPa in spite of much thicker capsule shells ranging from 60 to 130 nm in the case of pea protein capsules. The critical difference between these two systems could be related to the essentially milder conditions employed by Ye *et al.* for the synthesis of their capsules. Instead of ultrasound energy density of 200 W cm^−2^ applied by us for 1 minute, these authors have used a total acoustic power of 160 W for only 30 seconds. As a result, the shell of the obtained capsules was probably composed rather not of pea protein molecules regularly cross-linked by intermolecular cysteine bridges but probably formed by small protein aggregates with hydrodynamic radius between 5 and 93 nm^27^ attached to the interface and perhaps occasionally interconnected by ultrasound treatment. This very weak and inhomogeneous crosslinking density in the capsule shells is in the good agreement with their low stiffnesses reported in this paper.^27^ Whereas the thickness increased by more than factor 2 the stiffness of these microcapsules just displayed an insignificant scattering within error limits. In contrast, microcapsules with evenly interconnected shells should demonstrate an increasing stiffness as a function of the shell thickness. For example, polyelectrolyte microcapsules with an electrostatic attraction of oppositely charged polyelectrolyte layers in the shell showed a clear increase depending linearly on the second degree of the scaled dimensionless shell thickness.^28^

The counterintuitive finding of a reduction of the effective Young’s modulus due to presence of a nanoparticulate component in the microcapsule shell can be explained by the effects of stress concentration. If the nanoparticles are present in concentrations below the percolation threshold, which we assume to be the case in our system, rather than reinforcing the membrane, the particles act as local heterogenities. The large difference between the particles Young’s modulus and the Young’s modulus of the surrounding polymeric matrix will result in local stress concentration at the particle–matrix interface which could lead to crack formation at lower deformations and consequently a reduction of shell stiffness.

Similar behavior was also observed by Kolesnikova and coworkers.^29^ They synthesized polyelectrolyte microcapsules and embedded ZnO nanoparticles in their shell. The capsules without ZnO NPs in the shell with a size of 10.2 ± 0.2 μm and a shell thickness of 32.2 ± 2.3 nm exhibited a Young’s modulus of 580 ± 286 MPa. Embedding three or four layers of ZnO NPs led to an increase of the diameters to 13.1 ± 0.3 and 14.8 ± 0.3 μm, respectively whereas the shell thickness grew to 96.8 ± 7.2 and 103.7 ± 4.5 nm, respectively. The corresponding Young’s moduli revealed significantly lower values as compared with the nanoparticle-free shells – 27.1 ± 8.8 MPa and 30.5 ± 5.9 MPa, respectively. Within framework of the Reissner model, the reported decrease of the Young’s modulus is a direct consequence of the increasing number of ZnO layers in the capsules shell accompanied by a significant increase in its thickness. A weak increase in the Young’s modulus observed for the highest number of ZnO layers can be explained by the attainment of high “critical” concentration of nanoparticles in the shell, at which the interactions between them can contribute to the shell stiffness.^30^ The reported results^29^ are, on the first glance, quite comparable with our findings. However, this apparent analogy should be treated with caution, taking into account the specific layer-by-layer morphology of the polyelectrolyte microcapsules as well as the high error limits for the data given in ref. 29.

Another system comparable to the cross-linked BSA microcapsules was described by de Loubens and coworkers.^31^ They examined the mechanical properties of human serum albumin (HSA) microcapsules, which were synthesized by cross-linking of HSA with terephthaloyl chloride using droplets of HSA solution in a water-in-oil emulsion as templates. As the emulsification was carried out by simple stirring at 625 rpm, the obtained capsules had sizes in the range from 50 to 500 μm and thus were approximately one order of magnitude larger than ones studied in the paper at hand. The SFM measurements were performed using the colloidal probe technique and the obtained Young’s moduli varied from 20 kPa to 2 MPa increasing strongly with the size of microcapsules. In contrast to the papers considered above, de Loubens *et al.* used the Hertz model^32^ for the calculation of the Young’s modulus of microcapsules. According to this approach, the Young’s modulus is related to the applied force *F* as follows:2
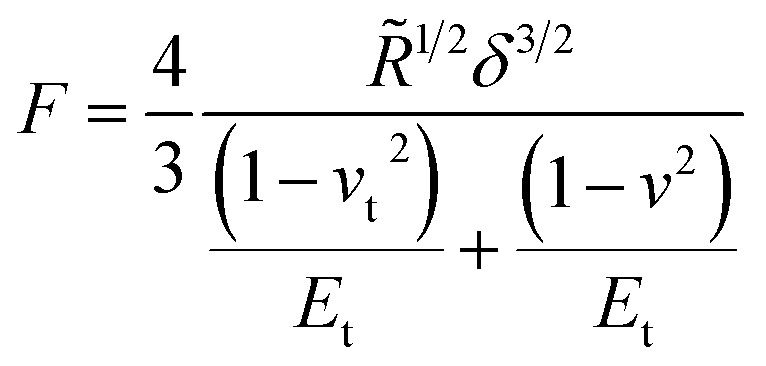
with 
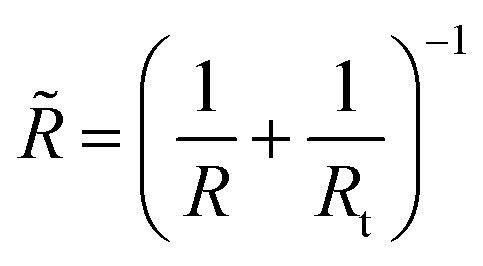
where *E*_t_ is the Young’s modulus of the tip with the colloidal probe and *E* − of the capsule, while *ν*_t_ = *ν* = 0.5 are the Poisson ratios of the tip and capsule. *R*_t_ and *R* are the radius of the tip and the capsule, respectively and *δ* is the indentation.

This formalism, however, does not account for the membrane-like character of the capsules shell and for its parameters like thickness or crosslinking density. Considering these factors allowed authors^31^ to explain, at least qualitatively, the observed behavior. The interfacial cross-linking reaction leading to the formation of a microcapsules shell was occurring at the boundary of two phases, and the protein as a water-soluble reagent was enclosed inside a confined aqueous droplet. Therefore, the volume of this droplet as well as the concentration of the protein solution, should determine the amount of the protein accessible for the reaction and its transfer rate to the interface. These parameters are, in turn, of key importance for the characteristics of microcapsules shell. The corresponding estimations yielded^31^ the thicknesses from 2 μm for the smallest microcapsules obtained on the basis of a 20 wt% HSA solution to over 20 μm for the large capsules with an enclosed 10 wt% HSA solution. On the other hand, the evaluation model used^31^ led to the dependence of the Young’s modulus on the size (radius) of microcapsules, which has never been reported formerly in the literature. This dependence was especially remarkable for the capsules prepared with the lowest concentration of HSA within the droplets of protein solution. As the product of the HSA concentration in the droplet and its volume (proportional to the third degree of the droplet radius) is a total mass of the HSA inside, the protein amount in the shell is also increased with the increase of these two parameters. Thus, this finding means that the Young’s modulus of the investigated capsules should be dependent on the amount of the HSA in the shell. In the reality, however, the Young’s modulus is an intensive property of a material and is therefore independent of its mass. The encountered contradiction is, with high probability, related to the limited applicability of the Hertz model to the microcapsules investigated by de Loubens *et al.*^31^

Both models, Hertz and Reissner, are applicable in the elastic regime, which corresponds to the small deformations. The Reissner model becomes more appropriate as the *h*/*R* ratio decreases, while the Hertz model is employed for soft cell-like shells in spherical approximation.^33^ In addition, compared to the Reissner model, the Hertz model takes into account the shape of the tip. Nevertheless, using the Hertz model to investigate the force–deformation curves may result in an underestimation of the rigidity. Eid and coworkers found a discrepancy between the values calculated using the Hertz model and the values obtained with the aid of other theories being up to three orders of magnitude higher for the Reissner model. At the same time, the values obtained according to the Reissner model were closer to the values obtained by other experimental techniques.^34^

As mentioned above, the Hertz model, in contrast to the Reissner model, does not take into account the shell thickness of the capsules. This feature as well as the clear-cut distinctions between the preparation technique as well as the size and the shell thickness for the HSA capsules^31^ and for the BSA microcapsules investigated in this work resulted in several order-of-magnitude differences in the corresponding Young’s moduli.

## Conclusions

In the shell of the protein capsules, the BSA molecules are cross-linked *via* intermolecular disulfide bonds induced by the ultrasound treatment. The shell of the composite capsules does not consist of cross-linked BSA molecules since the adsorption of BSA on the silica nanoparticles leads to conformational changes in the protein prohibiting the cross-linking. Therefore, the resulting microcapsules represent the droplets of an oil-in-water Pickering emulsion with essentially lower Young’s modulus. The mechanical properties of protein and composite protein-mineral microcapsules were investigated by means of SFM and the Young’s moduli calculated on the basis of the Reissner model were compared. The obtained results reveal that the cross-linked microcapsules offer higher resistance to the elastic deformation than non-cross-linked composite microcapsules, in spite of higher rigidity of nanoparticulate building blocks and enhanced thickness of capsule shell. On the other hand, the increase of the NP concentration in the capsule shell leads to an increase of the Young’s modulus.

## Conflicts of interest

There are no conflicts to declare.

## Supplementary Material

RA-012-D2RA04330K-s001
