# Supplementary material for: Nanodeformations of microcapsules: comparing the effects of cross-linking and nanoparticles
Source: RSC Adv. 2022 Aug 24;12(37):24140–5. doi: 10.1039/d2ra04330k (PMC9400586; doi:10.1039/d2ra04330k)
Supplement: RA-012-D2RA04330K-s001 [file RA-012-D2RA04330K-s001.pdf]

Electronic Supplementary Material (ESI) for RSC Advances.
This journal is © The Royal Society of Chemistry 2022

Supporting Information

Nanodeformations of Microcapsules: Comparing the Effects of Cross-linking and Nanoparticles

Ulrike Doering,[a] Dmitry Grigoriev,[*b] Tino Riske,[c] Andreas Fery[c] and Alexander Böker[b]

a. University of Potsdam, Institute of Chemistry, Karl-Liebknecht-Str. 24-25, 14476 Potsdam, Germany
b. Fraunhofer Institute for Applied Polymer Research IAP, Geiselbergstr. 69, 14476 Potsdam, Germany
c. Leibniz-Institute for Polymer Research Dresden e.V., Hohe Str. 6, 01069 Dresden, Germany

Email: Dmitry Grigoriev* – dmitry.grigoriev@iap.fraunhofer.de

* Corresponding Author

Nanocompression measurements with SFM

The nanocompression measurements were carried out with SFM using a tipless cantilever to deform the microcapsules. A microscope image of this measurement is shown in Fig. S1.

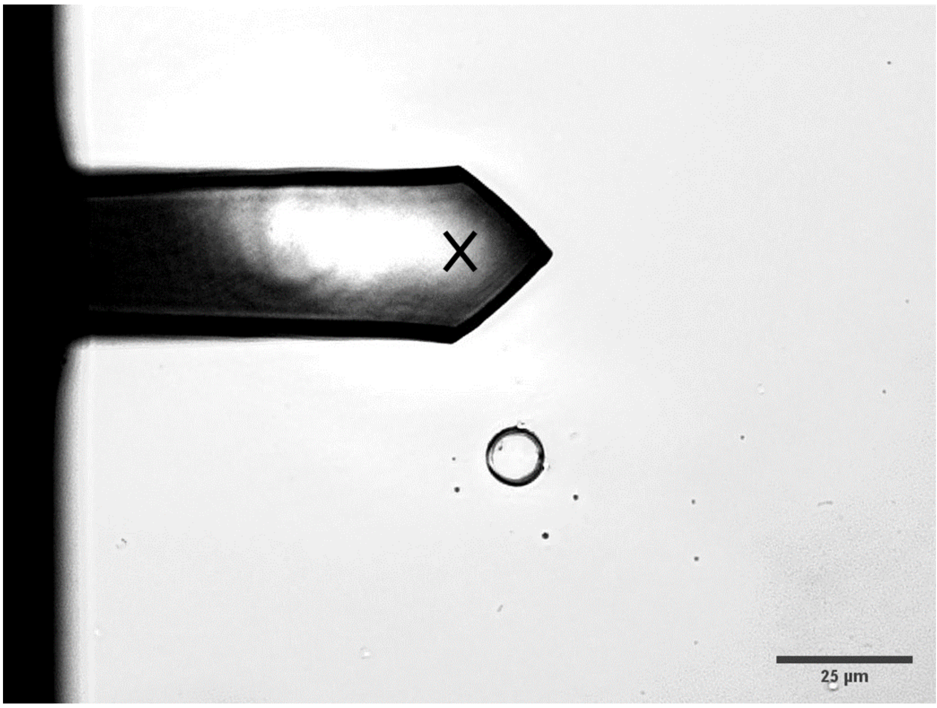

Fig. S1. Microscope image of the cantilever and a microcapsule loaded with Miglyol. The X denotes the part of the cantilever, which lower side was pressed to the microcapsule in course of nanocompression experiments enabling a homogeneous deformation of the microcapsule shell.

Determination of the wall thickness empty BSA microcapsules

Scanning force microscopy (SFM) was used to determine the wall thickness of empty cross-linked BSA microcapsules that were loaded with toluene. The result is displayed in Fig. S2.

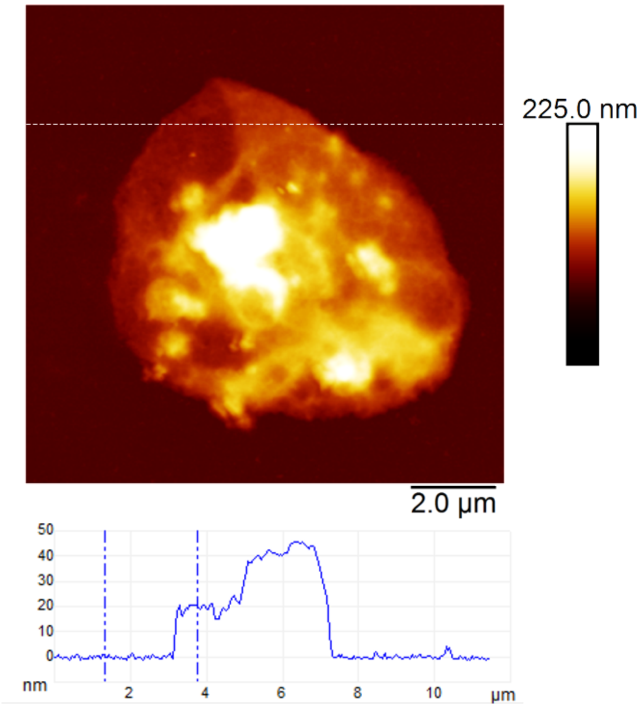

Fig. S2. SFM height image and height profile diagram of an empty and dried BSA microcapsule.

The SFM height image shows a microcapsule dried at standard laboratory conditions. The sample was prepared from a microcapsule emulsion, which was allowed to stay for 2 weeks only covered with Parafilm, the core of the capsules made of volatile toluene was evaporated and only the thin protein based capsule shells remained. A small amount of this aqueous suspension was deposited on a silicon wafer for further evaporation of the water, so that the sample could be examined with SFM. The microcapsules exhibit a spheroidal morphology with many folds, wrinkles and craters on the shell but the round shape is still mainly intact after the process of evaporation and drying. The height profile diagram shows two different heights at approximately 20 nm and 40 nm. The height image illustrates that along the line where the height profile was measured, the protein shell is folded on the top right causing a larger height value of 40 nm. In the area with a height of 20 nm the shell is not folded but taking into account that the dried flattened microcapsule still has two layers of the protein shell due to spherical shape in its original state it means that the protein microcapsule has a shell thickness of 10 nm.
